# The impact of Ramadan fasting on Fetuin-A level in type 2 diabetes mellitus

**DOI:** 10.1016/j.heliyon.2021.e06773

**Published:** 2021-05-15

**Authors:** Dante S. Harbuwono, Brama I. Sazli, Farid Kurniawan, Budiman Darmowidjojo, Sukamto Koesnoe, Dicky L. Tahapary

**Affiliations:** a Division of Endocrinology, Department of Internal Medicine, Dr. Cipto Mangunkusumo National Referral Hospital, Faculty of Medicine Universitas Indonesia, Jl. Diponegoro No.71, Central Jakarta, 10430, Indonesia; b Metabolic, Cardiovascular and Aging Cluster, The Indonesian Medical Education and Research Institute, Faculty of Medicine Universitas Indonesia, Jl. Diponegoro No.71, Central Jakarta, 10430, Jakarta, Indonesia; c Division of Allergy and Immunology, Department of Internal Medicine, Dr. Cipto Mangunkusumo National Referral Hospital, Faculty of Medicine Universitas Indonesia, Jl. Diponegoro No.71, Central Jakarta, 10430, Indonesia

**Keywords:** Ramadan fasting, Diabetes mellitus, Fetuin-A, Insulin resistance, Glycoprotein

## Abstract

**Background/Aims:**

Ramadan fasting creates changes in lifestyle, causing biochemical alterations that affect glucose metabolism and insulin sensitivity. This study aims to assess the impact of Ramadan fasting on glycemic control and Fetuin-A, a glycoprotein that affects insulin resistance, in patients with type 2 diabetes mellitus (T2DM).

**Materials and methods:**

This was a prospective study done among 37 patients with T2DM from Internal Medicine Polyclinic in a hospital in Jakarta, Indonesia. Anthropometric data as well as Hemoglobin A1c (HbA1c), Fasting Blood Glucose (FBG), and Fetuin-A levels of the subjects were measured in three time points: before, during, and after Ramadan fasting. A bivariate analysis was done to see the effect of Ramadan fasting on those parameters.

**Results:**

Ramadan fasting reduced Fetuin-A levels [median (minimum–maximum), 5.35 (2.91–7.81) vs. 3.22 (2.35–5.60) mg/dl; p = 0.039] four weeks after the end of Ramadan compared to pre-Ramadan. After two weeks of Ramadan fasting, we found a significant reduction in body weight, BMI, FBG, and HbA1c levels which rebounded to baseline level after Ramadan.

**Conclusion:**

Ramadan fasting was associated with a significant decrease in Fetuin-A level post Ramadan.

## 1 Introduction

The impact of Ramadan fasting on the health of people with diabetes mellitus (DM) has become a great interest with the increasing worldwide prevalence of DM, especially in countries where the majority of the population are Muslims. Home of the largest Muslim population in the world, Indonesia has reported a dramatic increase in the prevalence of DM which reached 10.9% in 2018 [1]. This became of great interest as previous studies reported that the majority of DM patients decided to fast during Ramadan [2, 3, 4].

Ramadan fasting is the act of restraining oneself from eating, drinking, taking medications and/or smoking between dawn and sunset every year for one lunar month [3]. These changes in term of food intake, physical activity, and overall lifestyle might potentially affect the metabolic control of DM patients [5]. Despite the inconsistencies and heterogenicity between studies [6, 7], our group has previously reported that Ramadan fasting slightly improved overall metabolic profile and anthropometric measurements among type 2 diabetes melitus (T2DM) patients with relatively low incidence of hypoglycaemia [8].

It is important to note that in addition to inconsistencies between studies regarding the impact of Ramadan fasting on the metabolic and clinical profile of DM patients, only limited data are available studying the impact of Ramadan fasting on molecular markers of insulin resistance, the main pathogenesis of T2DM. Fetuin-A is a glycoprotein produced primarily by hepatic cells and adipocytes [9] that has a role in insulin resistance by binding to insulin receptor tyrosine kinase in muscles and fat. Epidemiologic studies have consistently shown an increase of Fetuin-A level in patients with obesity, metabolic syndrome, and T2DM [10, 11, 12, 13, 14].

In summary, the impact of Ramadan fasting on the metabolic and clinical outcome of DM patients might be on the slightest, however, its impact on subclinical level has rarely been assessed. Our study aims to assess the impact of Ramadan fasting on Fetuin-A level, a glycoprotein associated with insulin resistance.

## 2 Materials and Methods

This prospective observational study was conducted among T2DM patients who fasted during Ramadan at the Metabolic Disorder, Cardiovascular and Aging Laboratory of The Indonesia Medical and Education Research Institute (IMERI), Faculty of Medicine Universitas Indonesia (FMUI). This study was part of The Diabetes and Ramadan Study Project 2019 held by the Division of Endocrinology and Metabolism, Internal Medicine Department, FMUI, Jakarta, Indonesia. Detailed methods of the Ramadan study projects have been published elsewhere [15]. Ethics approval was obtained from the Health Research Ethics Committee of Faculty of Medicine, Universitas Indonesia as stated in letter no. 0550/UN2.F1/ETIK/2019. T2DM patients at the Metabolic and Endocrine Clinic Dr. Cipto Mangunkusumo National Referral Hospital, a tertiary care hospital in Indonesia, who fasted during Ramadan month 2019 were recruited consecutively. The diagnosis of T2DM was established based on previous history of T2DM and/or HbA1c level of ≥6.5%.

The inclusion criteria were type 2 DM patients aged 40–70 years who fasted for a minimum of 14 days and consented to participate in this study. Subjects with cardiovascular disease, kidney disease, severe liver disease, chronic gastrointestinal disease, autoimmune disease, and history of hospital admission due to severe hypo- or hyperglycemia one month prior to Ramadan were excluded from the study. Changes of fetuin-A level throughout Ramadan were the primary outcomes of this study. The sample size was calculated using the formula for the average of two independent populations, which yielded a minimum of 30 subjects.

### 2.1 Data collection

Data collection which included questionnaires, physical examinations, and blood samples were performed in three consecutive time frames, which were 2–4 weeks before Ramadan (T0), after a minimum of 14 days of fasting (T1), and 4 weeks after the end of Ramadan (T2). Basic characteristics were collected through a standardized questionnaire that was filled out by the field recruitment officer in a face-to-face interview session with the participants. The questionnaire included data such as age, sex, days of fasting, history of hypertension and dyslipidemia. Blood pressure measurement was done using automatic blood pressure monitors (GEA Medical® type SH-2A High Meter 2 M) in a sitting position. Body height was measured using a portable stadiometer (GEA Medical®) and waist circumference was measured using an ergonomic circumference measuring tape at the middle point between the last palpable rib and the top of iliac crest. Bodyweight was measured in kilograms using Tanita MC780MA portable bioimpedance analyzer (BIA). BMI was calculated by using the ratio of weight (kg) to the square of height (m). BMI of 23–24.9 kg/m^2^ was considered as overweight, while BMI of ≥25 kg/m^2^ was considered as obese according to WHO Asia Pacific criteria [16]. Recommendation during observation were not given to the participants in order to minimize the potential sources of bias. However, counselling related to risks that may be faced during fasting is regularly done 1–2 months before Ramadhan.

Blood samples were withdrawn after at least 10 h of fasting. Fasting blood glucose (FBG), HbA1c, total cholesterol, high-density lipoprotein (HDL-C), low-density lipoprotein (LDL-C), triglyceride, liver transaminase enzyme, and creatinine were measured using a standardized laboratory method. Fetuin-A was analyzed using ELISA kit (Duoset; R&D Systems, Minneapolis, MN).

### 2.2 Statistical analysis

Data analysis was performed using SPSS for Mac version 20. Baseline characteristics of the study participants were presented descriptively. All normally distributed data were presented in mean [Standard deviation (SD)]and median [Interquartilerange (IQR)]. We analyzed the changes of the study parameters before (T0), during (T1), and after Ramadan fasting (T2) using paired t-test between T0 and T1, T1 and T2, as well as T0 and T2 for normal data distribution. Wilcoxon test was done for non-normal data distribution. P value of <0.05 was considered significant.

## 3 Results

Thirty-seven subjects were included in this study. Most subjects were women (54.1%) with a median age of 48 (44–53) years old. The baseline characteristics of the subjects are available in Table 1.

**Table 1:** Baseline characteristics of subjects.

Parameters	n = 37
Age (years), mean (SD) median (IQR)	53,05 (6,884) (53 (47,50–58,50))
Women, n (%)	20 (54.1)
Fasting duration (days), mean (SD), median (IQR)	17,58 (3,028), 17 (15–17)
Hypertension, n (%)	14 (37.8)
Dyslipidemia, n (%)	13 (35.1)
Systolic Blood Pressure (mmHg), mean (SD), median (IQR)	130,72 (15,87), 130 (120–140)
Diastolic Blood Pressure (mmHg), Mean (SD), median IQR	80,42 (9,44), 80 (70–90)
Body Weight (kg), mean (SD), median (IQR)	65.41 (11.46), 65,8 (56,55–72,63)
Obese, n (%)	7 (18.9)
Waist Circumference, mean (SD) median (IQR)	89,38 (11,47), 88 (81,55–97,75)

Abbreviations:

Min-max: minimum – maximum.

SD: Standard deviation.

After 14-days of fasting, we observed a reduction in Fetuin A level (from 5.35 (2.91–7.81) to 3.68 (2.33–5.89), Table 2), but it did not reach statistical significance. However, further reduction was observed at 4-weeks after the end of Ramadan, reaching a lower level [3.22 (2.35–5.60) mg/dl; p = 0.039, Table 2] in comparison to its baseline pre-Ramadan level. The changes in parameters can be seen in Table 2.

**Table 2:** Changes in parameters over time.

Parameter	2–4 weeks before fasting (T0)	Minimum 14 days of fasting (T1)	4 weeks after fasting (T2)	P value (T0-T1)	P value (T1-T2)	P value (T0-T2)
Fetuin-A (mg/ml)	5.35 (2.91–7.81)	3.68 (2.33–5.89)	3.22 (2.35–5.60)	0.217	0.637	**0.039**
Body weight (kg)	65.41 (11.46)	64.13 (11.30)	65.66 (11.95)	**<0.001**	**<0.001**	0.375
BMI (kg/m^2^)	26.77 (4.52)	26,24 (4.42)	26.76 (4.65)	**<0.001**	0.985	**<0.001**
FBG (mg/dl)	150 (114–195)	131 (104–165)	154 (124–194)	**0.013**∗	**0.003**	0.519
HbA1C (%)	8.1 (6.85–10.5)	7.9 (6.85–9.40)	8 (6.85–8.95)	**0.004**	**<0.001**	0.366

Abbreviations: SD: standard deviation; BMI: Body Mass Index; FBG: Fasting Blood Glucose; HbA1C: Haemoglobin A1C.

Data for T0, T1, and T2 are presented as mean (SD) for normal data distribution, or median (minimum-maximum) for non-normal data distribution.

Analysis: paired t-test.

The reduction of Fetuin A level after 14 days of fasting was in line with a parallel significant decrease of body weight (p < 0.001), BMI (p < 0.001), FBG (p = 0.013), and HbA1c (p = 0.004). However, four weeks after the end of Ramadan, in contrast to the further reduction we observed in the Fetuin A level, all of those metabolic and clinical parameters rebounded to their baseline levels.

∗ Wilcoxon test.

The reduction of Fetuin A level after 14 days of fasting was in line with a parallel significant decrease of body weight (p < 0.001), BMI (p < 0.001), FBG (p = 0.013), and HbA1c (p = 0.004). However, four weeks after the end of Ramadan, in contrast to the further reduction we observed in the Fetuin A level, all of those metabolic and clinical parameters rebounded to their baseline levels (Table 2).

## 4 Discussion

This prospective observational study was performed among patients with T2DM who practiced Ramadan fasting consecutively for a minimum of 14 days. In this study, Fetuin-A levels were significantly lower in the 4 weeks after Ramadan fasting compared to baseline. In addition, we found a significant decrease in bodyweight, BMI, FBG, and HbA1c after 2 weeks of fasting, which rebounded 4 weeks after Ramadan fasting.

As increase in Fetuin-A levels are associated with the increase of insulin resistance, the decrease levels of Fetuin-A during and after Ramadan fasting found in our study might show the beneficial effect of Ramadan fasting in the cellular levels. Lifestyle interventions, such as dietary changes and physical activity, have been shown to decrease Fetuin-A levels [17, 18]. Previous studies also showed that calorie restriction and bodyweight reduction, either in DM or non-DM patients, can reduce the concentration of serum Fetuin-A [19, 20]. Therefore, the decrease of body weight observed in our study might partially explain the decline in Fetuin-A level. Along with bodyweight reduction, the number or the size of adipocytes as one main producer of Fetuin-A might also decrease. However, this hypothesis still needs to be confirmed by measurement of body fat composition. It would also be interesting to analyze the association between total calorie intake and physical activity changes that are often practiced during Ramadan fasting, and the serum Fetuin-A level.

In contrast to the rebounded metabolic and clinical parameters 4 weeks after the end of Ramadan fasting, interestingly, the serum levels of Fetuin-A still decreased, even statistically significant compared to baseline. This might be caused by a delayed effect of body weight decrease during fasting or continuous beneficial response of Ramadan fasting. Either way, it might suggest the presumably long-term effect of Ramadan fasting. A similar finding also observed by Blüher et al [21] in obese subjects who underwent long-term weight loss intervention which displayed a continued decline of Fetuin-A levels despite partial weight regain.

Previous studies have shown a similar weight-decreasing effect of Ramadan fasting [22, 23, 24, 25]. Maintenance of body weight in one individual is mainly affected by the energy balance concept, the amount of calories intake from food, and the output of calories utilized for basal metabolism and daily physical activities [22, 23]. As shown by most previous studies, the total energy expenditure did not differ significantly between Ramadan and non-Ramadan periods in the subjects committed to practice Ramadan practice [24]. This left the possibility of presumably lower calorie intake during Ramadan fasting as the cause for decreasing body weight. This hypothesis is supported by the result observed from a previous study [25], although there are still some conflicting data [26, 27].

Another mechanism thought to play a role in the weight loss during Ramadan fasting is the more efficient fat utilization observed during fasting [28] A study by Syam et al showed that the decrease in body weight during Ramadan fasting was significantly affected by the decrease in body fat composition, especially visceral fat [29]. The decrease in body weight could lead to normalization of BMI in obese T2DM patients and potentially affect glycemic control positively, which is one of the targets that should be achieved by DM patients [30].

The improved glycemic control during Ramadan fasting in our study, as shown by FBG and HbA1c levels, supports findings from previous studies [8, 31, 32, 33, 34, 35]. However, these findings have to be interpreted carefully. The difference in the timing of FBG measurement before and during Ramadan fasting might contribute to this observed improvement. Ideally, the measurement has to be performed at the same time, which is in the morning after a minimum of 8 h fasting, as soon as the Ramadan month ended. Since HbA1c reflects the glycemic control from previous 2–3 months, the improvement of HbA1c might not reflect the effect of Ramadan fasting in our study. The use of fructosamine, which reflect the average plasma glucose over a period of 2–3 weeks, may be a more reliable indicator of glycemic control for short periods of fasting in Ramadan [36, 37].

In our study, in concordance with previous studies [8, 15], we did not find an increased incidence of hypoglycemia or hyperglycemia event during Ramadan fasting (data not shown). The inclusion of only low to moderate risk diabetic patients in our and previous studies might be the reason for this finding. The IDF-DAR guideline also only recommends these groups of patients to perform Ramadan fasting safely [38].

This study is the first to observe the impact of Ramadan fasting on T2DM patients toward Fetuin-A level in association with other clinical and metabolic parameters. Observation and measurement of subjects before, during, and after Ramadan fasting could be considered as the strength of this study although with limited number of subjects. However, Fetuin-A level and other parameters were significantly decreased after Ramadhan fasting could be affected due to the naturalistic observation condition from this study. In addition, we treated the same attention to all participants and this can be applied in primary care setting in Indonesia to give safety recommendation for patients with T2DM who choose to fast during Ramadhan. Moreover, the length of Ramadhan is only 30 days annually, thus extended intermittent fasting might have a beneficial changes in T2DM patients due to the changes of feeding habits and calorie restriction during fasting.

## 5 Conclusion

In summary, Ramadan fasting is associated with a persistent reduction in Fetuin-A level despite a temporary improvement in other metabolic parameters. Future studies with more patients and multivariate analysis are needed in order to analyse the inflammatory markers and insulin resistance levels this would be needed to get a clearer picture of the effect of Ramadan fasting on Fetuin-A levels and its associations with those parameters.

## Declarations

### Author contribution statement

Dante Saksono Harbuwono and Dicky Levenus Tahapary: Conceived and designed the experiments; Analyzed and interpreted the data; Wrote the paper.

Brama Ihsan Sazli: Conceived and designed the experiments; Performed the experiments; Analyzed and interpreted the data; Contributed reagents, materials, analysis tools or data; Wrote the paper.

Budiman Darmowidjojo and Sukamto Koesnoe: Conceived and designed the experiments; Analyzed and interpreted the data.

Farid Kurniawan: Performed the experiments; Contributed reagents, materials, analysis tools or data; Wrote the paper.

### Funding statement

This work was supported by 10.13039/501100006378Universitas Indonesia (NKB-4110/UN2.RST/HKP.05.00/2020).

### Data availability statement

Data included in article/supplementary material/referenced in article.

### Declaration of interests statement

The authors declare no conflict of interest.

### Additional information

No additional information is available for this paper.
